# Alterations of Striatal Subregions in a Prion Protein Gene V180I Mutation Carrier Presented as Frontotemporal Dementia With Parkinsonism

**DOI:** 10.3389/fnagi.2022.830602

**Published:** 2022-04-15

**Authors:** Zhongyun Chen, Jinghong Ma, Li Liu, Shuying Liu, Jing Zhang, Min Chu, Zhen Wang, Piu Chan, Liyong Wu

**Affiliations:** ^1^Department of Neurology, Xuanwu Hospital, Capital Medical University, Beijing, China; ^2^National Clinical Research Center for Geriatric Diseases, Beijing, China

**Keywords:** frontotemporal dementia with parkinsonism, prion protein gene, striatum, dopamine, positron emission tomography

## Abstract

**Objective:**

To explore the roles of striatal subdivisions in the pathogenesis of frontotemporal dementia with parkinsonism (FTDP) in a patient resulting from prion protein gene (PRNP) mutation.

**Methods:**

This patient received clinical interviews and underwent neuropsychological assessments, genetic testing, [^18^F]-fluorodeoxyglucose positron emission tomography ([^18^F]-FDG PET)/MRI, and [^18^F]-dihydrotetrabenazine positron emission tomography ([^18^F]-DTBZ PET)/CT. Region-of-interest analysis was conducted concerning metabolism, and dopamine transport function between this patient and 12 controls, focusing on the striatum subregions according to the Oxford-GSK-Imanova Striatal Connectivity Atlas.

**Results:**

A 64-year-old man initially presented with symptoms of motor dysfunction and subsequently behavioral and personality changes. FTDP was initially suspected. Sequence analysis disclosed a valine to isoleucine at codon 180 in *PRNP*. Compared to controls, this patient had a severe reduction (> 2SD) of standard uptake value ratio (SUVR) in the limbic and executive subregions but relative retention of metabolism in rostral motor and caudal motor subregions using [^18^F]-FDG PET/MRI, and the SUVR decreased significantly across the striatal in [^18^F]-DTBZ PET/CT, especially in the rostral motor and caudal motor subregions.

**Conclusion:**

The alteration of frontal striatal loops may be involved in cognitive impairment in FTDP, and the development of parkinsonism in FTDP may be primarily due to the involvement of the presynaptic nigrostriatal loops in *PRNP V180I* mutation.

## Introduction

Frontotemporal dementia with parkinsonism (FTDP) is a group of degenerative disorders that can occur sporadically or in families. Mutations in the genes encoding the microtubule-associated protein tau (*MAPT*) and progranulin (*PGRN*) o chromosome 17 have been linked to familial FTDP (Boeve and Hutton, 2008). There has been an increase in the number of cases with a clinical diagnosis of FTDP that were shown to be genetic prion diseases caused by prion protein gene (*PRNP*) mutations (Nitrini et al., 2001; Woulfe et al., 2005; Jansen et al., 2010; Bernardi et al., 2014). However, the underlying mechanism behind this remains elusive.

The striatum, aside from its fundamental movement function evidenced by parkinsonian deficits, is involved in processing of closely related non-motor, cognitive and reward information. Previous research found that the caudate was the most vulnerable to lesion in sporadic amyotrophic lateral sclerosis–frontotemporal dementia (FTD) continuum patients associated with cognitive decline with FTD features (Masuda et al., 2016). Therefore, we hypothesize that alterations in striatal regions may be related to the mechanisms of cognitive and motor impairment in FTDP. In recent years, multiple imaging modalities have been used to explore the pathogenesis of the disease. For example, [^18^F]-fluorodeoxyglucose positron emission tomography ([^18^F]-FDG PET) is a technique for measuring changes in energy metabolism that reflect underlying cellular events. [^18^F]-dihydrotetrabenazine positron emission tomography ([^18^F]-DTBZ PET) reflects structural brain changes caused by pathology in the nigrostriatal dopamine system. Previous studies using [^18^F]-N-3-fluoropropyl-2β-carboxymethoxy-3β-(4-iodophenyl)-nortropane positron emission tomography ([^18^F]-FP-CIT PET). in patients with Parkinson’s disease (PD) and parkinsonism based on the anatomical subregions of the striatum found that the loss of dopamine transporters differed across the striatal subregions among diseases and can be used for disease differentiation (Oh et al., 2012; Sung et al., 2017; Kong et al., 2020). However, anatomical subregions may not provide the best annotation of the striatum. Tziortzi et al. (2014) classified the striatum into functional subregions based on anatomical links between the striatum and the cortex, and the homogeneity of dopamine release was significantly higher than the structural subregion. It remains unclear whether genetic prion diseases develop FTDP as a result of striatal functional area involvement.

To address this issue, we performed a series of assessments of metabolic and dopamine transporter function in a genetic prion disease caused by *PRNP* V180I mutation presented with FTDP, with a focus on the functional regions of the striatum.

## Methods

### Ethics Statement

The study was approved by the Ethics Committees of the Xuanwu Hospital of Capital Medical University and carried out in accordance with the principles of the Helsinki Declaration. Each patient or their guardian provided written informed consent.

### Study Design

A patient with clinical diagnosis of behavioral variant FTD and progressive parkinsonism confirmed to be genetic prion disease (*PRNP* V180I) was enrolled. Thereafter, 12 gender- and age-matched healthy controls and 3 cases with PD who fulfilled the UK Parkinson’s Disease Society Brain Bank clinical diagnostic criteria admitted to the Department of Neurology at Xuanwu Hospital between 1 January 2019 and 31 January 2021 were recruited to our cohort.

The patient with genetic prion disease underwent genetic analysis, cerebrospinal fluid (CSF) 14-3-3 protein levels and prion real-time quaking-induced conversion (RT-QuIC) testing, electroencephalography (EEG) monitoring, and brain magnetic resonance imaging (MRI). Genomic DNA was extracted from fresh peripheral blood leukocytes, and whole-exome sequencing (WES) libraries were generated using the Agilent SureSelect Human All Exon V6 Kit (Agilent Technologies, Santa Clara, CA, United States). CSF protein 14-3-3 levels and RT-QuIC were detected at the National Reference Laboratory for Human Prion Diseases, CDC, China, according to the working procedures described previously (Gao et al., 2011; Xiao et al., 2019). Two-hour EEG monitoring was performed using a 21-lead EEG transducer (Micromed, Italy). The EEG electrodes were placed according to the International 10–20 system. MRI was performed at 3.0 T (Erlangen, Germany) with the following sequences: T1 weighted image (T1WI), T2 weighted image (T2WI), fluid-attenuated inversion recovery (FLAIR), diffusion-weighted imaging (DWI), and apparent diffusion coefficient (ADC) values.

All the subjects underwent clinical interviews and examination, including neuropsychological assessments and cerebral [^18^F]-FDG PET/MRI examinations. Six controls and all the cases with PD underwent [^18^F]-DTBZ PET/computed tomography (CT).

### Neuroimaging Acquisition and Preprocessing

#### [^18^F]-Fluorodeoxyglucose Positron Emission Tomography/Magnetic Resonance Imaging Acquisition and Preprocessing

The [^18^F]-FDG PET/MRI scans were carried out with the help of a GE Signa PET/MR 3.0 Tesla scanner (GE Healthcare, Milwaukee, WI, United States). A 30-min dynamic scan was acquired approximately 45 min after intravenous injection of 3.7 MBq/kg of ^18^F-FDG. Attenuation correction, scattering correction, random correction, decay correction, and dead-time correction were performed on the PET data. Corrected PET data were obtained using a time-of-flight, point spread function, ordered subset expectation maximization (TOF-PSF-OSEM) algorithm with 4 iterations and 16 subsets. Finally, all images were spatially normalized to the PET Montreal Neurological Institute (MNI) brain space template, scaled, averaged, and then smoothed using 8-mm full width at half maximum (FWHM) Gaussian kernel using SPM12 (Statistical Parametric Mapping 12)^1^ running under MATLAB 7.11 (Mathworks Inc., Sherborn, MA, United States) on the CentOS 6.5. The intensity of [^18^F]-FDG PET/MRI scans was normalized using the entire cerebellar reference region to generate standard uptake value ratio (SUVR) images.

#### [^18^F]-Dihydrotetrabenazine Positron Emission Tomography/Computed Tomography Acquisition and Preprocessing

The [^18^F]-DTBZ PET/CT scans were performed on a Gemini GXL 16 PET/CT scanner (Philips, Amsterdam, the Netherlands) in three-dimensional iterative mode. Ninety minutes after injecting approximately 250 MBq of [^18^F]-DTBZ radiotracer ([^18^F]-AV133), 15 min of brain scans were obtained. Using the ^18^F-AV133 PET template, the florbenzaine images were spatially normalized to the standard atlas. The target regions were the assigned regions of interest based on the atlas (caudate, anterior and posterior putamen, and the entire striatum). The SUVR was calculated by calculating the ratio of tracer activity in the target region relative to the occipital cortex as the reference region.

#### Analysis at the Striatal Subregion Level

The striatum was subdivided using the Oxford-GSK-Imanova Striatal Connectivity Atlas, a probabilistic atlas that divides the striatum into seven subregions based on white matter connections to cortical regions (Tziortzi et al., 2014). To begin, the striatum was divided into four subregions, each of which was linked to the frontal, parietal, occipital, and temporal lobes. The frontal subregion is further subdivided into four subregions (limbic, executive, rostral motor, and caudal motor). Limbic subregion is linked to the anterior orbital gyrus, posterior orbital gyrus, medial orbital gyrus, gyrus rectus, and subcallosal gyrus-ventral anterior cingulate; executive subregion is linked to the rostral superior and middle frontal gyri and the dorsal prefrontal cortex; rostral motor subregion is linked to the caudal portions of lateral and medial superior gyrus as well as the caudal middle and inferior frontal gyri; caudal motor subregion is linked to the precentral gyrus that corresponds functionally to the primary motor cortex (Area 4) and the caudal premotor area (Caudal area 6).

### Statistical Analysis

Analyses were performed using SPSS version 22.0 (IBM, Armonk, NY, United States). Continuous data are represented as the mean ± SD. Two or more standard deviations from the mean between groups were considered statistically significant.

## Results

### Case Presentation

**FIGURE 1:**
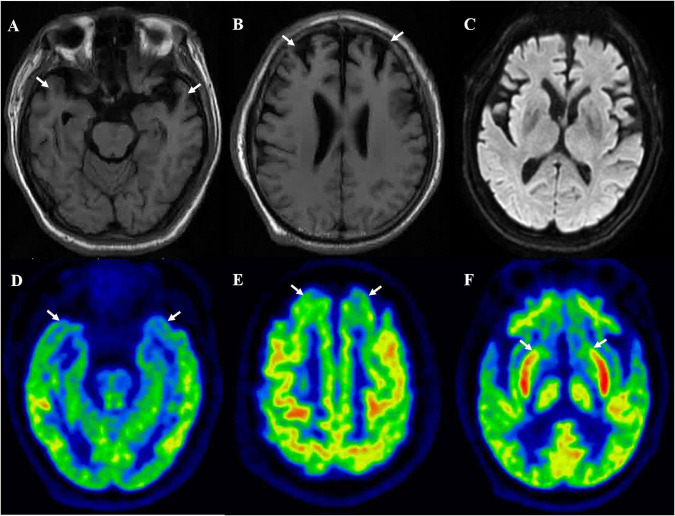
Brain MRI suggested bilateral frontal **(B)** and temporal **(A)** lobes atrophy, and no hyperintensity was detected in diffusion-weighted imaging **(C)**. [^18^F]-FDG-PET/MRI suggested bilateral frontal lobe **(E)**, temporal lobe **(D)**, and caudate nucleus **(F)** hypometabolism.

A 64-year-old man presented with initial symptoms of limb rigidity, bradykinesia. In the following months, he developed involuntary limb tremors and gait abnormality, but was still able to walk independently; these symptoms can be partially alleviated by levodopa. One year after the onset of symptoms, he developed apathy, behavior abnormality, loss of sympathy or empathy, hyperorality, and disinhibition, and had difficulty in daily activities. The symptoms of parkinsonism also became aggravated; he had severe bilateral lower extremity rigidity with inability to flex, leading to a bed-bound state, and was accompanied by fecal incontinence and sweating. He showed no improvement to antiparkinson and dystonia-relieving drugs. He had no family history concerning similar neurodegenerative diseases. Neurological examination 26 months after onset revealed akinetic mutism, marked rigidity in all of the extremities, bilateral hyporeflexia, and extensor Babinski signs with the Hoehn and Yahr of stage V. Cognitive assessment (mini-mental state examination and Montreal Cognitive Assessment scores) did not cooperate. Brain MRI displayed bilateral frontotemporal lobe atrophy (Figures 1A,B) and no hyperintensity was detected in diffusion-weighted imaging or fluid attenuation inversion recovery imaging (Figure 1C). FTDP was initially suspected. Genomic DNA extracted from peripheral blood was used to identify mutations associated with FTDP. It turned out that the patient carried a point mutation of valine to isoleucine at codon 180, as well as an MM polymorphism at codon 129 in *PRNP*. No mutations were detected in FTD-related genes, such as *MAPT*, *GRN*, *TARDBP*, *FUS*, *C9orf72*, *VCP*, *CHMP2B*, *SQSTM1*, and *TBK1*.

Ancillary tests related to piron diseases were implemented further, and CSF analyses revealed a negative 14-3-3 protein assay. The CSF prion RT-QuIC assay was negative. EEG showed non-specific slow waves but lacking a definite periodism.

### [^18^F]-Fluorodeoxyglucose Positron Emission Tomography Analysis

The [^18^F]-FDG PET showed diffuse cortical and subcortices hypometabolism (Figures 1D–F). Compared to controls, this patient had a severe reduction of SUVR in the limbic subregion (25.9%) and executive subregion (27.7%), but the motor subregion did not differ between this patient and controls using functional subdivision. Detailed data are shown in Table 1.

**TABLE 1:** Comparison of the striatal functional distribution in this case and controls using [^18^F]-FDG-PET/MRI.

Striatal subregion	[^18^F]-FDG-PET/MRI (SUVR value)	
	Controls (*N* = 12)	Case with *PRNP* mutation
Limbic subregion	1.43 ± 0.16	1.06^†^
Executive subregion	1.37 ± 0.18	0.99^†^
Rostral motor subregion	1.37 ± 0.18	1.20
Caudal motor subregion	1.41 ± 0.20	1.26
Parietal subregion	1.48 ± 0.19	1.45
Occipital subregion	1.34 ± 0.17	1.36
Temporal subregion	1.02 ± 0.12	0.94

*^†^Indicated values in patient with V180I mutation are 2SD lower than the mean value for the controls in the same brain regions.*

### [^18^F]-Dihydrotetrabenazine Positron Emission Tomography Analysis

**FIGURE 2:**
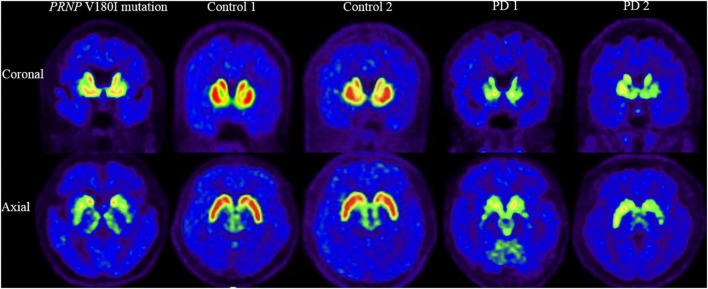
[^18^F]-DTBZ-PET/CT imaging of the VMAT2 distribution in patient with *PRNP* V180I mutation and controls/cases with PD. The same slice is presented for the case and two controls and two cases with PD. This patient with *PRNP* V180I mutation has obvious symmetric dopamine decline of the bilateral putamen, predominantly in the posterior part. While Controls 1 and 2 have normal dopamine metabolism, asymmetric decline of vesicular transporter availability with a posterior-to-anterior gradient can be seen in PD 1 and PD 2.

[^18^F]-DTBZ PET revealed reduced radioactivity in bilateral putamen, predominantly in the posterior part. Asymmetric decline of vesicular transporter availability with a posterior-to-anterior gradient can be seen in cases with PD (Figure 2). The functional subdivision analysis suggested the SUVR across the striatal decreased (> 2SD) significantly, with a reduction of 40.5% in rostral motor region and 47.7% in caudal motor subregion compared to controls. Detailed data are provided in Table 2.

**TABLE 2:** Comparison of the striatal functional distribution in this case and controls/cases with PD using [^18^F]-DTBZ-PET/CT.

Striatal subregion	[^18^F]-DTBZ-PET/CT (SUVR value)		
	Controls (*N* = 6)	PD (*N* = 3)	Case with *PRNP* mutation
Limbic subregion	3.35 ± 0.11	2.49 ± 0.07	2.40^†^
Executive subregion	2.96 ± 0.08	2.08 ± 0.18	1.97^†^
Rostral motor subregion	2.42 ± 0.10	1.66 ± 0.17	1.44^†^
Caudal motor subregion	2.83 ± 0.16	1.64 ± 0.15	1.48^†^
Parietal subregion	3.01 ± 0.14	1.63 ± 0.26	1.61^†^
Occipital subregion	2.60 ± 0.31	2.00 ± 0.21	2.24
Temporal subregion	–	–	–

*^†^Indicated values in patient with V180I mutation are 2SD lower than the mean value for the controls in the same brain regions.*

## Discussion

In this study, we present the first case of a *PRNP* V180I carrier presenting with the clinical syndrome of FTDP. Furthermore, we investigated the pathogenesis of FTDP related to striatal subdivision in this patient using multiple imaging modalities. Our findings showed that this case had hypometabolism in cognition-related limbic and executive subregions, while the motor-related rostral and caudal motor areas were mismatched for metabolism and dopamine transport function. These findings suggest that disruption of frontal striatal loops may be involved in cognitive impairment in FTDP, and that disruption of nigrostriatal loops may be a strong argument in favor of *PRNP* mutation-associated parkinsonism, providing a more complete understanding of the mechanisms underlying striatal-associated FTDP in subjects with *PRNP* mutations.

Genetic prion diseases carrying V180I mutation have been widely reported in East Asia. Patients carrying this mutation usually have atypical clinical and laboratory features (Qina et al., 2014). Previous studies have reported that V180I mutation can mimic Alzheimer’s disease (AD) (Bagyinszky et al., 2019a) or dementia with Lewy bodies (DLB) (Tomizawa et al., 2020). FTD or FTDP is a rare genetic prion disease phenotype. To date, 20 point mutations and 3 insertional mutations in *PRNP* have been reported to be associated with this phenotype (Supplementary Table 1). Patients with the FTD phenotype often lack the typical ancillary findings of prion disease. Of the published prion patients with the FTD/FTDP phenotype, it is roughly estimated that no patients have periodic sharp-wave complexes on EEG, three quarters had no hyperintensity on MRI, and two-thirds were negative for CSF 14-3-3 protein (Nitrini et al., 2001; Hall et al., 2005; Woulfe et al., 2005; Clerici et al., 2008; Giovagnoli et al., 2008; Alzualde et al., 2010; Bernardi et al., 2010, 2014; Jansen et al., 2010, 2011; Kumar et al., 2011; Beck et al., 2013; Cupidi et al., 2013; McKnight et al., 2013; San Millán et al., 2013; Riudavets et al., 2014; Mano et al., 2016; Oldoni et al., 2016; Kenny et al., 2017; Ghetti et al., 2018; Sun et al., 2018; Takayanagi et al., 2018; Bagyinszky et al., 2019b; Di Fede et al., 2019; Priemer et al., 2019; Townley et al., 2020).

The striatum is widely acknowledged to have connections with the cortex and to play an important role in the striato-cortical circuitry, which has been characterized by several functionally segregated subcircuits that are anatomically different but functionally adjacent. In this study, a connectivity-based (CB) functional striatum atlas was used to provide optimal subdivision of the striatum. In the CB functional striatum atlas, the frontal lobe connections dominate the total volume of the striatum and are the main factor influencing the functional organization of the striatum (Tziortzi et al., 2014). We presumed that the reduction of SUVR in the limbic and executive subregions of the striatum may be secondary to the hypometabolism of the frontal lobes through the frontal-striatal loops in this patient.

This case showed a severe hypometabolism in the caudate nucleus and a relatively preserved metabolism in the putamen in [^18^F]-FDG PET, which is distinguished from PD or other parkinsonism, such as hypermetabolism of the putamen in PD and metabolic reductions of the putamen in multiple system atrophy (Eckert et al., 2005; Akdemir et al., 2014; Meles et al., 2020). The symmetrical low intake of the putamen using [^18^F]-DTBZ PET also differs in patients with PD, who generally have asymmetrical presynaptic dopaminergic synapse selectively degenerated, causing the decrease of dopaminergic uptake with a posterior to the anterior gradient in the striatum (Nandhagopal et al., 2009). Putamen-related motor circuits are well known to be responsible for motor functions, and putamen dysfunction correlates well with the onset of cardinal motor symptoms and motor severities. It is surprising that this patient had severe motor symptoms while metabolism in this area was relatively preserved. Further, functional subdivision analysis suggested the SUVR in motor subregions decreased significantly using dopamine transporter function imaging while relatively preserved metabolism on metabolic imaging. This mismatch pattern of metabolism and dopamine transport function of motor subregions suggests that parkinsonism caused by *PRNP* mutation may be mainly associated with presynaptic degeneration of the nigrostriatal pathway. This finding contradicts the conventional perception that parkinsonism in prion diseases is generally regarded to be postsynaptic structural deterioration caused by spongiform degeneration of basal ganglia (Maltête et al., 2006). Although the application of [^123^I] FP-CIT in a few cases of genetic prion disease also found altered presynaptic dopamine function, none of these studies evaluated the metabolism of the striatum (Malek et al., 2017; Baiardi et al., 2020; Tomizawa et al., 2020). Of course, no response to levodopa therapy observed in our patient could be explained by post-synaptic dysfunction of the nigrostriatal pathway. There may also be deterioration of the postsynaptic nigrostriatal pathway in this patient due to lower SUVR in motor subregions than in controls, although no significant difference was reached. Indeed, a combination of presynaptic and postsynaptic cell loss in the substantia nigra system has been observed in pathological studies of patients with sporadic prion diseases (Vital et al., 2009).

There were some limitations to this study. First, the negative results of Creutzfeldt-Jakob disease (CJD) supportive investigation (CSF 14-3-3, EEG, MRI, and RT-QuIC) and the lack of pathology data to support a diagnosis of prion disease in this patient do not rule out the potential of an incidental V180I combination, as V180I carriers have only a weak to moderate elevated risk of developing prion disease (Minikel et al., 2016). Second, because genetic prion diseases are extremely rare, the results were hampered by small sample size. Third, the enrolled patient was in a relatively advanced stage of the disease with severe clinical manifestations and lack of imaging of dynamic changes. Hence, more research into striatal subdivisions in larger genetic prion diseases with FTDP or parkinsonism is required.

In summary, the frontal striatal loops may involved in cognitive impairment of FTDP and the movement dysfunction of FTDP may be primarily due to the involvement of the presynaptic nigrostriatal loops. Measuring metabolism and dopaminergic changes in a functionally defined striatal region could provide a more sensitive tool for detecting *PRNP*-associated specific striatal changes.

## Data Availability Statement

The raw data supporting the conclusions of this article will be made available by the authors, without undue reservation.

## Ethics Statement

The studies involving human participants were reviewed and approved by the Ethics Committees of the Xuanwu Hospital of Capital Medical University. The patients/participants provided their written informed consent to participate in this study.

## Author Contributions

LW: study concept and design. JM, LL, SL, ZC, MC, and ZW: acquisition of data analysis and interpretation of data. ZC: drafting of the manuscript. JM, LL, SL, MC, JZ, ZW, PC, and LW: critical revision of the manuscript for important intellectual content. All authors read and approved the final manuscript.

## Conflict of Interest

The authors declare that the research was conducted in the absence of any commercial or financial relationships that could be construed as a potential conflict of interest.

## Publisher’s Note

All claims expressed in this article are solely those of the authors and do not necessarily represent those of their affiliated organizations, or those of the publisher, the editors and the reviewers. Any product that may be evaluated in this article, or claim that may be made by its manufacturer, is not guaranteed or endorsed by the publisher.

## Footnotes

1
http://www.fil.ion.ucl.ac.uk/spm

## Abbreviations

FTDP: frontotemporal dementia with parkinsonism
*PRNP*: prion protein gene
*MAPT*: microtubule-associated protein tau
*PGRN*: progranulin
PD: Parkinson’s disease
CB: connectivity-based
SUVR: standard uptake value ratio
[^18^F]-FDG PET: [^18^F]-fluorodeoxyglucose positron emission tomography
[^18^F]-DTBZ PET: [^18^F]- dihydrotetrabenazine positron emission tomography.

## References

[B1] Akdemir Ü Tokçaer A. B. Karakuş A. Kapucu L. (2014). Brain 18F-FDG PET imaging in the differential diagnosis of parkinsonism. *Clin. Nucl. Med.* 39 e220–e226. 10.1097/RLU.0000000000000315 24321825

[B2] Alzualde A. Indakoetxea B. Ferrer I. Moreno F. Barandiaran M. Gorostidi A. (2010). A Novel PRNP Y218N mutation in gerstmann-sträussler-scheinker disease with neurofibrillary degeneration. *J. Neuropathol. Exp. Neurol.* 69 789–800. 10.1097/NEN.0b013e3181e85737 20613639

[B3] Bagyinszky E. Kang M. J. Pyun J. Giau V. V. An S. S. A. Kim S. (2019a). Early-onset Alzheimer’s disease patient with prion (PRNP) p.Val180Ile mutation. *Neuropsychiatr. Dis. Treat* 15 2003–2013. 10.2147/NDT.S215277 31410005 PMC6645694

[B4] Bagyinszky E. Yang Y. Giau V. V. Youn Y. C. An S. S. A. Kim S. (2019b). Novel prion mutation (p.Tyr225Cys) in a Korean patient with atypical Creutzfeldt-Jakob disease. *Clin. Interv. Aging* 14 1387–1397. 10.2147/CIA.S210909 31447551 PMC6683949

[B5] Baiardi S. Rizzi R. Capellari S. Bartoletti-Stella A. Zangrandi A. Gasparini F. (2020). Gerstmann-Sträussler-Scheinker disease (PRNP p.D202N) presenting with atypical parkinsonism. *Neurol. Genet.* 6:e400. 10.1212/NXG.0000000000000400 32274419 PMC7112137

[B6] Beck J. Poulter M. Hensman D. Rohrer J. D. Mahoney C. J. Adamson G. (2013). Large C9orf72 hexanucleotide repeat expansions are seen in multiple neurodegenerative syndromes and are more frequent than expected in the UK population. *Am. J. Hum. Genet.* 92 345–353. 10.1016/j.ajhg.2013.01.011 23434116 PMC3591848

[B7] Bernardi L. Anfossi M. Gallo M. Geracitano S. Colao R. Puccio G. (2010). Prion protein insertion in a family affected by frontotemporal dementia associated to the PSEN1 V412I mutation. *Clin. Neuropathol.* 29:186.

[B8] Bernardi L. Cupidi C. Frangipane F. Anfossi M. Gallo M. Conidi M. E. (2014). Novel N-terminal domain mutation in prion protein detected in 2 patients diagnosed with frontotemporal lobar degeneration syndrome. *Neurobiol. Aging* 35:2657. 10.1016/j.neurobiolaging.2014.06.006 25022973

[B9] Boeve B. F. Hutton M. (2008). Refining frontotemporal dementia with parkinsonism linked to chromosome 17: introducing FTDP-17 (MAPT) and FTDP-17 (PGRN). *Arch. Neurol.* 65 460–464. 10.1001/archneur.65.4.460 18413467 PMC2746630

[B10] Clerici F. Elia A. Girotti F. Contri P. Mariani C. Tagliavini F. (2008). Atypical presentation of Creutzfeldt-Jakob disease: the first Italian case associated with E196K mutation in the PRNP gene. *J. Neurol. Sci.* 275 145–147. 10.1016/j.jns.2008.06.036 18706660

[B11] Cupidi C. Bernardi L. Frangipane F. Clodomiro A. Colao R. Puccio G. (2013). Identification of the novel PRNP gene mutation PRO39LEU in patients affected by frontotemporal dementia. *Func. Neurol.* 28:18.

[B12] Di Fede G. Catania M. Atzori C. Moda F. Pasquali C. Indaco A. (2019). Clinical and neuropathological phenotype associated with the novel V189I mutation in the prion protein gene. *Acta Neuropathol. Commun.* 7:1. 10.1186/s40478-018-0656-4 30606247 PMC6317215

[B13] Eckert T. Barnes A. Dhawan V. Frucht S. Gordon M. F. Feigin A. S. (2005). FDG PET in the differential diagnosis of parkinsonian disorders. *Neuroimage* 26 912–921. 10.1016/j.neuroimage.2005.03.012 15955501

[B14] Gao C. Shi Q. Tian C. Chen C. Han J. Zhou W. (2011). The epidemiological, clinical, and laboratory features of sporadic Creutzfeldt-Jakob disease patients in China: surveillance data from 2006 to 2010. *PLoS One* 6:e24231. 10.1371/journal.pone.0024231 21904617 PMC3164193

[B15] Ghetti B. Bonnin J. Garringer H. Richardson R. Epperson F. Fong J. (2018). Neurofibrillary tau pathology and PrP amyloidosis are associated with the PRNP Q160X nonsense mutation. *J. Neuropathol. Exp. Neurol.* 77 529–530. 10.1007/s00401-021-02336-w 34128081 PMC8270882

[B16] Giovagnoli A. R. Di Fede G. Aresi A. Reati F. Rossi G. Tagliavini F. (2008). Atypical frontotemporal dementia as a new clinical phenotype of Gerstmann-Straussler-Scheinker disease with the PrP-P102L mutation. description of a previously unreported Italian family. *Neurol. Sci.* 29 405–410. 10.1007/s10072-008-1025-z 19030774

[B17] Hall D. A. Leehey M. A. Filley C. M. Steinbart E. Montine T. Schellenberg G. D. (2005). PRNP H187R mutation associated with neuropsychiatric disorders in childhood and dementia. *Neurology* 64 1304–1306. 10.1212/01.WNL.0000156911.70131.06 15824374

[B18] Jansen C. Parchi P. Capellari S. Strammiello R. Dopper E. G. P. Van Swieten J. C. (2011). A second case of Gerstmann-Sträussler-Scheinker disease linked to the G131V mutation in the prion protein gene in a Dutch patient. *J. Neuropathol. Exp. Neurol.* 70 698–702. 10.1097/NEN.0b013e3182270c54 21760536

[B19] Jansen C. Parchi P. Capellari S. Vermeij A. J. Corrado P. Baas F. (2010). Prion protein amyloidosis with divergent phenotype associated with two novel nonsense mutations in PRNP. *Acta Neuropathol.* 119 189–197. 10.1007/s00401-009-0609-x 19911184 PMC2808512

[B20] Kenny J. Woollacott I. Koriath C. Hosszu L. Adamson G. Rudge P. (2017). A novel prion protein variant in a patient with semantic dementia. *J. Neurol. Neurosurg. Psychiatry* 88 891–892. 10.1136/jnnp-2017-315577 28572272 PMC5629930

[B21] Kong Y. Zhang C. Liu K. Wagle Shukla A. Sun B. Guan Y. (2020). Imaging of dopamine transporters in Parkinson disease: a meta-analysis of (18) F/(123) I-FP-CIT studies. *Ann. Clin. Transl. Neurol.* 7 1524–1534. 10.1002/acn3.51122 32794655 PMC7480930

[B22] Kumar N. Boeve B. F. Boot B. P. Orr C. F. Duffy J. Woodruff B. K. (2011). Clinical characterization of a kindred with a novel 12-octapeptide repeat insertion in the prion protein gene. *Arch. Neurol.* 68 1165–1170. 10.1001/archneurol.2011.187 21911696 PMC3326586

[B23] Malek N. Jampana R. Grosset D. G. (2017). Rare case of atypical parkinsonism: why family history is important. *Scott. Med. J.* 62 159–162. 10.1177/0036933017727966 29192564

[B24] Maltête D. Guyant-Maréchal L. Mihout B. Hannequin D. (2006). Movement disorders and Creutzfeldt-Jakob disease: a review. *Parkinsonism Relat. Disord.* 12 65–71. 10.1016/j.parkreldis.2005.10.004 16364674

[B25] Mano K. K. Matsukawa T. Mitsui J. Ishiura H. Tokushige S. Takahashi Y. (2016). Atypical parkinsonism caused by Pro105Leu mutation of prion protein: a broad clinical spectrum. *Neurol. Genet.* 2:e48. 10.1212/NXG.0000000000000048 27066585 PMC4817902

[B26] Masuda M. Senda J. Watanabe H. Epifanio B. Tanaka Y. Imai K. (2016). Involvement of the caudate nucleus head and its networks in sporadic amyotrophic lateral sclerosis-frontotemporal dementia continuum. *Amyotroph. Lateral Scler. Frontotemporal Degener.* 17 571–579. 10.1080/21678421.2016.1211151 27684890

[B27] McKnight K. Herron B. Turkington J. Haffey S. Mead S. Mcmonagle P. (2013). Inherited prion disease due to 5-octapeptide repeat insertion. *J. Neurol. Sci.* 333 e334–e335. 10.1212/01.wnl.0000267642.41594.9d 17709704

[B28] Meles S. K. Renken R. J. Pagani M. Teune L. K. Arnaldi D. Morbelli S. (2020). Abnormal pattern of brain glucose metabolism in Parkinson’s disease: replication in three European cohorts. *Eur. J. Nucl. Med. Mol. Imaging* 47 437–450. 10.1007/s00259-019-04570-7 31768600 PMC6974499

[B29] Minikel E. V. Vallabh S. M. Lek M. Estrada K. Samocha K. E. Sathirapongsasuti J. F. (2016). Quantifying prion disease penetrance using large population control cohorts. *Sci. Transl. Med.* 8:322ra9. 10.1126/scitranslmed.aad5169 26791950 PMC4774245

[B30] Nandhagopal R. Kuramoto L. Schulzer M. Mak E. Cragg J. Lee C. S. (2009). Longitudinal progression of sporadic Parkinson’s disease: a multi-tracer positron emission tomography study. *Brain* 132 2970–2979. 10.1093/brain/awp209 19690093

[B31] Nitrini R. Da Silva L. S. Rosemberg S. Caramelli P. Carrilho P. E. Iughetti P. (2001). Prion disease resembling frontotemporal dementia and parkinsonism linked to chromosome 17. *Arq. Neuropsiquiatr.* 59 161–164. 10.1590/s0004-282x2001000200001 11400017

[B32] Oh M. Kim J. S. Kim J. Y. Shin K. H. Park S. H. Kim H. O. (2012). Subregional patterns of preferential striatal dopamine transporter loss differ in Parkinson disease, progressive supranuclear palsy, and multiple-system atrophy. *J. Nucl. Med.* 53 399–406. 10.2967/jnumed.111.095224 22323779

[B33] Oldoni E. Fumagalli G. G. Serpente M. Fenoglio C. Scarioni M. Arighi A. (2016). PRNP P39L variant is a rare cause of frontotemporal dementia in italian population. *J. Alzheimers Dis.* 50 353–357. 10.3233/JAD-150863 26757195

[B34] Priemer D. Garringer H. Richardson R. Epperson F. Zanusso G. Ghetti B. (2019). Novel neuropathologic findings in the first american case of frontotemporal dementia associated with the T183A PRNP mutation. *J. Neuropathol. Exp. Neurol.* 78 569–570.

[B35] Qina T. Sanjo N. Hizume M. Higuma M. Tomita M. Atarashi R. (2014). Clinical features of genetic Creutzfeldt-Jakob disease with V180I mutation in the prion protein gene. *BMJ Open* 4:e004968. 10.1136/bmjopen-2014-004968 24838726 PMC4025468

[B36] Riudavets M. A. Sraka M. A. Schultz M. Rojas E. Martinetto H. Begué C. (2014). Gerstmann-sträussler-scheinker syndrome with variable phenotype in a new kindred with PRNP -P102L mutation. *Brain Pathol.* 24 142–147. 10.1111/bpa.12083 23944754 PMC8029128

[B37] San Millán B. Teijeira S. Rodriguez R. Yañez R. Navarro C. (2013). Gerstmann-Strãussler-Scheinker disease. description of the first case in galicia. *Clin. Neuropathol.* 32:541.

[B38] Sun Y. Xia M. Yang H. Zang W. Ma L. Wang S. (2018). Fatal familial insomnia preliminarily diagnosed as frontotemporal dementia: a case report and literature review. *Chin. J. Neurol.* 51 294–298.

[B39] Sung C. Lee J. H. Oh J. S. Oh M. Lee S. J. Oh S. J. (2017). Longitudinal decline of striatal subregional [(18)F]FP-CIT Uptake in Parkinson’s disease. *Nucl. Med. Mol. Imaging* 51 304–313. 10.1007/s13139-017-0481-x 29242724 PMC5721089

[B40] Takayanagi M. Suzuki K. Nakamura T. Hirata K. Satoh K. Kitamoto T. (2018). Genetic Creutzfeldt-Jakob disease with a glutamate-to-lysine substitution at codon 219 (E219K) in the presence of the E200K mutation presenting with rapid progressive dementia following slowly progressive clinical course. *Clin. Neurol.* 58 682–687. 10.5692/clinicalneurol.cn-001206 30369528

[B41] Tomizawa Y. Taniguchi D. Furukawa Y. (2020). Genetic Creutzfeldt-Jakob disease mimicking dementia with Lewy bodies: clinical and radiological findings. *J. Neurol. Sci.* 409:116604. 10.1016/j.jns.2019.116604 31805431

[B42] Townley R. A. Polsinelli A. J. Fields J. A. Machulda M. M. Jones D. T. Graff-Radford J. (2020). Longitudinal clinical, neuropsychological, and neuroimaging characterization of a kindred with a 12-octapeptide repeat insertion in PRNP: the next generation. *Neurocase* 26 211–219. 10.1080/13554794.2020.1787458 32602775 PMC7426006

[B43] Tziortzi A. C. Haber S. N. Searle G. E. Tsoumpas C. Long C. J. Shotbolt P. (2014). Connectivity-based functional analysis of dopamine release in the striatum using diffusion-weighted MRI and positron emission tomography. *Cereb. Cortex* 24 1165–1177. 10.1093/cercor/bhs397 23283687 PMC3977617

[B44] Vital A. Fernagut P. O. Canron M. H. Joux J. Bezard E. Martin-Negrier M. L. (2009). The nigrostriatal pathway in Creutzfeldt-Jakob disease. *J. Neuropathol. Exp. Neurol.* 68 809–815. 10.1097/NEN.0b013e3181abdae8 19535991

[B45] Woulfe J. Kertesz A. Frohn I. Bauer S. George-Hyslop P. S. Bergeron C. (2005). Gerstmann-Sträussler-Scheinker disease with the Q217R mutation mimicking frontotemporal dementia. *Acta Neuropathol.* 110 317–319. 10.1007/s00401-005-1054-0 16025285

[B46] Xiao K. Shi Q. Zhou W. Zhang B. Y. Wang Y. Chen C. (2019). T188K-Familial Creutzfeldt-Jacob disease, predominant among Chinese, has a reactive pattern in CSF RT-QuIC different from D178N-fatal familial insomnia and E200K-familial CJD. *Neurosci. Bull.* 35 519–521. 10.1007/s12264-019-00354-z 30838505 PMC6527617

