# Supplementary material for: Alterations of Striatal Subregions in a Prion Protein Gene V180I Mutation Carrier Presented as Frontotemporal Dementia With Parkinsonism
Source: Front Aging Neurosci. 2022 Apr 15;14:830602. doi: 10.3389/fnagi.2022.830602 (PMC9053668; doi:10.3389/fnagi.2022.830602)
Supplement: Supplementary file 1 [file Table_1.docx]

**Supplementary Table 1** *PRNP* mutations associated with FTD and FTDP

| Classification | Mutations list |
| --- | --- |
| FTD-associated *PRNP* mutations | P39L, G54S, P102L, P105L, A117V, G131V, R156C, Q160X, D167N, D178N-129MV, V180I, T183A, H187R, V189I, E196K, E200K, Q217R, Y218N, Y225C, Q227X, five, seven and twelve octapeptide repeat insertion |
| FTDP-associated *PRNP* mutations | G54S, P102L, P105L, G131V, D167N, D178N, V180I, T183A, H187R, V189I, E200K, Q217R, Y225C, Q227X, five and seven and twelve octapeptide repeat insertion |

FTD: frontotemporal dementia; FTDP: frontotemporal dementia with parkinsonism; *PRNP*: prion protein gene
